# LibSBMLSim: a reference implementation of fully functional SBML simulator

**DOI:** 10.1093/bioinformatics/btt157

**Published:** 2013-04-05

**Authors:** Hiromu Takizawa, Kazushige Nakamura, Akito Tabira, Yoichi Chikahara, Tatsuhiro Matsui, Noriko Hiroi, Akira Funahashi

**Affiliations:** Department of Biosciences and Informatics, Keio University, 3-14-1 Hiyoshi, Kouhoku-ku, Yokohama, Japan

## Abstract

**Motivation:** The Systems Biology Markup Language (SBML) is currently supported by >230 software tools, among which 160 support numerical integration of ordinary differential equation (ODE) models. Although SBML is a widely accepted standard within this field, there is still no language-neutral library that supports all features of SBML for simulating ODE models. Therefore, a demand exists for a simple portable implementation of a numerical integrator that supports SBML to enhance the development of a computational platform for systems biology.

**Results:** We implemented a library called libSBMLSim, which supports all the features of SBML and confirmed that the library passes all tests in the SBML test suite including those for SBML Events, AlgebraicRules, ‘fast’ attribute on Reactions and Delay. LibSBMLSim is implemented in the C programming language and does not depend on any third-party library except libSBML, which is a library to handle SBML documents. For the numerical integrator, both explicit and implicit methods are written from scratch to support all the functionality of SBML features in a straightforward implementation. We succeeded in implementing libSBMLSim as a platform-independent library that can run on most common operating systems (Windows, MacOSX and Linux) and also provides several language bindings (Java, C#, Python and Ruby).

**Availability:** The source code of libSBMLSim is available from http://fun.bio.keio.ac.jp/software/libsbmlsim/. LibSBMLSim is distributed under the terms of LGPL.

**Contact:** funa@bio.keio.ac.jp

**Supplementary information:** Supplementary data are available at *Bioinformatics* online.

## 1 INTRODUCTION

Understanding of the logic and dynamics of gene-regulatory and biochemical networks is a major challenge in systems biology. For this reason, there exist numerous software tools that can simulate ordinary differential equations (ODEs), and several efforts to standardize the model description of computational models have been made. One major model description language is the Systems Biology Markup Language (SBML), which is well accepted in the systems biology field (Hucka *et al.*, 2004).

Although there exist >230 SBML-supported software tools (Hucka *et al.*, 2011), there is still no language-neutral library that supports all SBML features and has been confirmed as passing all SBML tests. For example, few simulator completely support SBML Events, which is an element to describe a discontinuous change to a model with respect to time, number of particles, parameter values, etc. Supporting Events in the simulator requires some effort by developers if they are using one of the existing numerical integration libraries, because most of the integrators assume that the model is continuous.

Another element that is not well supported in SBML is the SBML AlgebraicRule element, which allows modelers to reduce the number of variables and to add a constraint to each variable. Therefore, supporting AlgebraicRule in the simulator involves far more than just implementing a numerical integrator for ODEs, because the simulator has to solve differential algebraic equations (DAEs), which requires an implementation to solve non-linear simultaneous equation numerically (or analytically) to determine the value of variables described in AlgebraicRule. This is also required when a simulator must support the ‘fast’ attribute on an SBML Reaction element.

The Delay function is a rarely supported SBML function in existing simulators, especially with a combination of Event elements. The Delay function allows a modeler to include biological processes having a delayed response. This function requires some additional modification to the ODE solver itself, to store calculated values that are used after a certain delay.

To carefully and quantitatively check the capability of each SBML elements on the simulator, it is recommended to run the test cases provided by the SBML test suite and validate each simulation results. SBML test suite consists of numerous combinations of SBML features in separate models with the expected results. The test results are also publicly released for existing software (http://sbml.org/Facilities/Database), and currently, there is still no language-neutral library that confirmed to pass all SBML tests.

## 2 APPROACH

To support all the functionality on SBML, we decided to implement a numerical integrator, non-linear simultaneous equation solver, abstract syntax tree (AST) manipulator and a handler for Events and Delays. The library is implemented in the C programming language because of C’s portability and performance. Supporting other programming languages will be achieved by providing language bindings.

## 3 METHODS

### 3.1 Numerical integrator

LibSBMLSim provides both explicit (Runge–Kutta) and implicit (backward Euler) methods (Hairer *et al.*, 1993) for numerical integration. Providing only explicit methods such as Runge–Kutta is not sufficient for biochemical simulation. Owing to the fact that there exist several reactions that proceed on different time scales, the system becomes stiff and requires implicit methods to solve the problem.
(1)
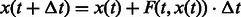

(2)
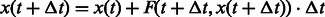



Equations (1) and (2) are examples of explicit (Euler) and implicit (backward Euler) methods, respectively. Here, 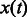 is a variable which is a function of time *t*, and 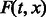 is defined by a differential equation. It is obvious from equation (2) that the integrator must solve this equation to find 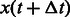 before the integration because both the left- and right-hand side of the equation contains the term 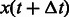. For this reason, we have developed a non-linear simultaneous equation solver. Furthermore, to improve the accuracy of integration, variable-step integrator (Runge–Kutta Fehlberg and Cash–Karp) and multistep methods are provided for both explicit (Adams–Bashforth) and implicit (Adams–Moulton and Gear) methods (Hairer *et al.*, 1993).

### 3.2 Non-linear simultaneous equation solver

To implement the above implicit methods, a non-linear simultaneous equation solver is implemented in our library. The non-linear simultaneous equation solver is also required to support the SBML AlgebraicRule element and the ‘fast’ attribute in the Reaction element. We used the *k*-dimensional Newton’s method to solve systems of non-linear simultaneous equations. Numerical differentiation is used to obtain the Jacobian matrix, and (for linear approximation) LU decomposition, Newton’s method, forward elimination and backward substitution are also implemented for this purpose.

### 3.3 AST manipulator

In SBML, ODEs are stored in the kineticLaw math element and can be obtained as an AST object (Supplemental Figure S1) by using libSBML (Bornstein *et al.*, 2008). Our library directly uses AST to handle all the functionality of SBML. When an SBML model is loaded, our library parses all ASTs and its related elements, and transforms the AST to suit with actual evaluation. Evaluation of AST is performed by converting AST to Reverse Polish notation (RPN). To improve the performance, we applied a stack architecture to avoid recursive calls and evaluation of each AST node. Using AST as a fundamental data structure of equations made our implementation straightforward. For example, if a model contains a FunctionDefinition element, the library will expand the AST node that calls the function to the actual content of the defined function (Supplemental Figure S2). This transformation of AST can be applied to support other SBML elements, such as piecewise functions. To support piecewise functions on AST, our library adds multiplication between each conditional expression and definition, and then combines them using addition and logical operators (NOT and AND) (See Supplemental Figure S3). The combination of the SBML Delay and EventAssignment element that is used to describe an Event to take place after a certain delay is also supported by this implementation.

### 3.4 Events and Delays

A SBML Event element is used to describe an instantaneous discontinuous change in a set of variables of any type (species quantity, compartment size or parameter value) when a triggering condition is satisfied (Hucka *et al.*, 2008). When a condition specified in Trigger changes from ‘false’ to ‘true’, an assignment described in EventAssignment is executed. If an Event contains a Delay element, then the assignment is executed after the delay described in the Delay element. From SBML Level 3 Version 1, Priority element and ‘persistent’ attribute on Trigger are introduced. Priority specifies the priority of Event, and ‘persistent’ attribute specifies whether the trigger expression should not have to be re-checked after it triggers. To support all functions of SBML Events, we implemented an event handler as follows: (i) Insert all Events that are related to a given Trigger to a queue. (ii) Calculate the priority of all Events in the queue. (iii) Move the Event that has the maximum priority value to the head of the queue and execute its EventAssignment. (iv) Reconfirm the Trigger of Events whose persistent flag is ‘false’, and remove from the queue if the Trigger is set to ‘false’ by the previous EventAssignment. (v) Insert any Event whose Trigger was changed from ‘false’ to ‘true’ by the above EventAssignment to the queue. (vi) Go back to procedure (ii) if there still exists an Event in the queue.

The Delay function is used to describe a delay differential equation (DDE) in a model. For example, when a model contains the differential equation 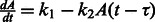, the simulator must use the value of 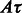 seconds before for the integration. A straightforward way to implement a DDE solver is to use a continuation method; however, in SBML, the delay variable 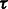 is not necessarily fixed, and thus the actual implementation in our library stores the values of all the variables during the simulation.

## 4 DISCUSSION

The aim of the current work is the development of a portable simulation library that fully supports SBML features. For this purpose, we at first focused on accuracy by a straightforward implementation, staying carefully within the specifications of SBML. Although there already exist elaborated and efficient solvers, numerical integrators including variable-step integrator and both explicit and implicit method are implemented from scratch because we wanted to reduce the number of dependent libraries as much as possible. We are planning to port this library on tablet computers and smartphones. SBML can also describe partial differential equations (PDEs), stochastic differential equations (SDEs) and discrete stochastic simulation models, which are not yet included in the SBML test suite. As yet libSBMLSim does not provide integrators for these types of models; a known limitation which we intended to address in our future development of the library.

## 5 CONCLUSION

LibSBMLSim is a portable simulation library that supports all the features of SBML Level 2 Version 4 and Level 3 Version 1. We confirmed that our library passed all 980 tests of the SBML test suite (version 2.0.2, See Supplemental Figure S4). The library does not depend on any third-party library except libSBML. Language bindings such as Java, C#, Python and Ruby are also included in the distribution.

*Funding*: This work was supported by a Grant-in-Aid for Scientific Research on Innovative Areas (23136513).

*Conflict of Interest*: none declared.

## Supplementary Material

Supplementary Data
