# Supplementary material for: LibSBMLSim: a reference implementation of fully functional SBML simulator
Source: Bioinformatics. 2013 Apr 5;29(11):1474–6. doi: 10.1093/bioinformatics/btt157 (PMC3661052; doi:10.1093/bioinformatics/btt157)
Supplement: Supplementary Data [file supp_29_11_1474__index.html]

LibSBMLSim: a reference implementation of fully functional SBML simulator — LibSBMLSim: a reference implementation of fully functional SBML simulator — Supplementary Data 

# LibSBMLSim: a reference implementation of fully functional SBML simulator

## Supplementary Data

files

**Files in this Data Supplement:**

- Supplementary Data - pdf file
