# Supplementary material for: LibSBMLSim: a reference implementation of fully functional SBML simulator
Source: Bioinformatics. 2013 Apr 5;29(11):1474–6. doi: 10.1093/bioinformatics/btt157 (PMC3661052; doi:10.1093/bioinformatics/btt157)
Supplement: Supplementary Data [file supp_btt157_supplement.pdf]

BIOINFORMATICS

Vol. 00 no. 00 2012
Pages 1

Supplemental figures

Hiromu Takizawa, Kazushige Nakamura, Akito Tabira, Yoichi Chikahara, Tatsuhiro Matsui, Noriko Hiroi and Akira Funahashi*

Department of Biosciences and Informatics, Keio University, 3-14-1 Hiyoshi Kouhoku-ku, Yokohama, Japan.

Received on XXXXX; revised on XXXXX; accepted on XXXXX

Associate Editor: XXXXXXX

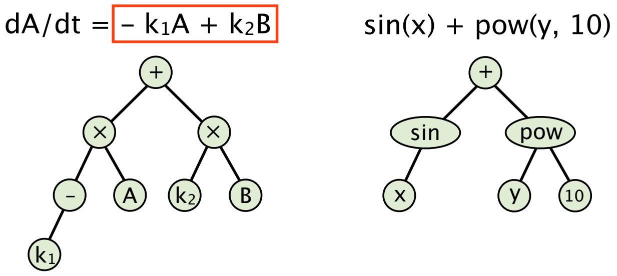

Fig. 1. Schematic diagram of AST and its content. Evaluation of AST can be done by achieving postorder traversal of AST and obtaining RPN.

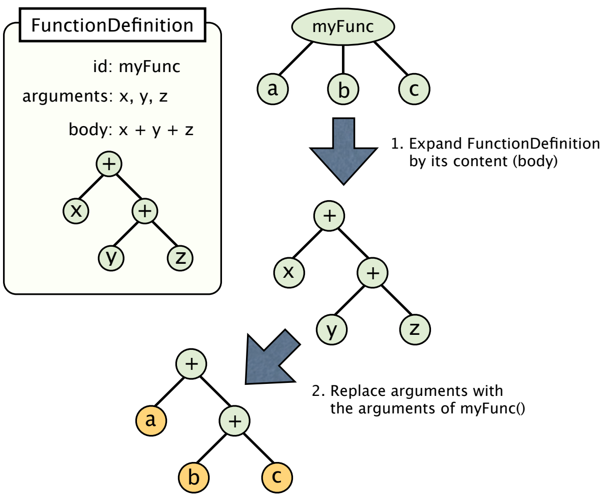

Fig. 2. Schematic diagram of how the FunctionDefinition element is expanded. At first, libSBMLSim will expand the AST node of FunctionDefinition to its content (step 1). Once the AST node is expanded, each arguments of AST will be replaced from the definition of FunctionDefinition (node x, y, z) to actual arguments (node a, b, c) (step 2).

*to whom correspondence should be addressed

© Oxford University Press 2012.

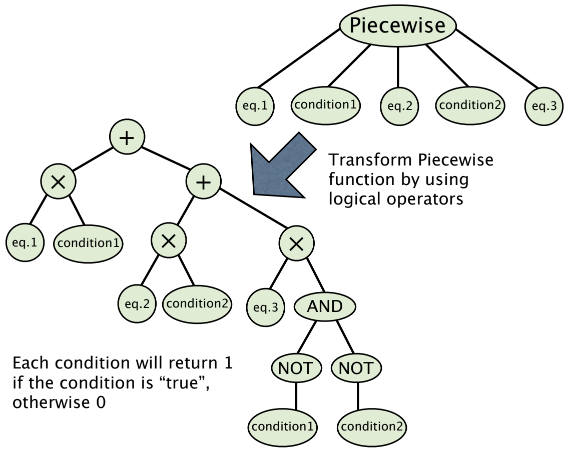

Fig. 3. Schematic diagram of how libSBMLSim handles piecewise function on AST. LibSBMLSim converts AST of piecewise function to a combination of multiplication and addition with logical operators (NOT and AND). Each condition (condition 1 and 2 in figure) will return 1 if the condition is "true", otherwise 0. With the combination of multiplication and addition, evaluating piecewise function as AST will become quite straightforward. There will be no branch operation while evaluating the function; the library will just traverse the AST and after the evaluation, expected result will be returned to the root node. This approach makes easy to implement the piecewise function, but on the other hand, there is a drawback that all the evaluation of conditions and equations ("eq." in the figure) will be applied, results to performance degradation and memory consumption.

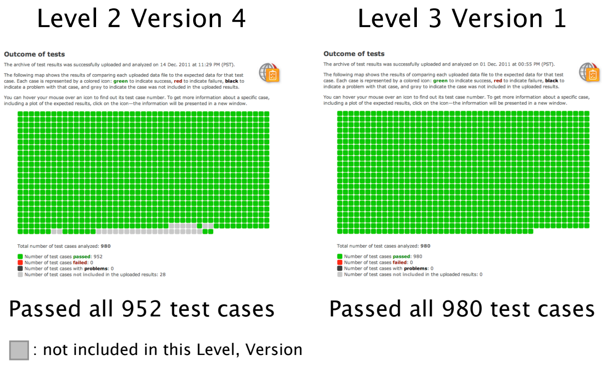

Fig. 4. Outcome of SBML test suite. SBML test suite version 2.0.2 provides 980 and 952 test cases for SBML Level 3 version 1 and SBML Level 2 version 4 respectively. SBML test suite provides a web-based service which accepts the simulation results of test cases as an input, and then displays the result of the tests as a matrix. Each cell represents the result of each test case. If the simulation result agrees with the expected result from SBML test case, then the cell will be colored as green. If the simulation result does not agree with the expected result, the cell will be colored as red. LibSBMLSim is confirmed to pass all the test cases both on SBML Level 3 version 1 and Level 2 version 4. LibSBML-5.2.0 is used as an SBML parser library for this test.
